# A qualitative study of ICU nurses assisting in Wuhan who suffered from workplace violence during the COVID‐19 outbreak

**DOI:** 10.1002/nop2.1984

**Published:** 2023-08-27

**Authors:** Guanhong Wu, Ying Lin, Xianghui Huang, Jianshan Zheng, Mengmeng Chang

**Affiliations:** ^1^ Gastroenterology Department Children's Hospital of Fudan University (Xiamen Branch), Xiamen Children's Hospital Xiamen China; ^2^ Neonatal Intensive Care Unit Children's Hospital of Fudan University (Xiamen Branch), Xiamen Children's Hospital Xiamen China; ^3^ Fujian Key Laboratory Of Neonatal Diseases Xiamen China

**Keywords:** coping methods, COVID‐19, ICU nurses, psychological experience, qualitative study, workplace violence

## Abstract

**Aim:**

To explore the psychological experience and coping methods of nurses exposed to workplace violence and to propose measures to prevent and control workplace violence and provide psychological assistance for health workers.

**Design:**

We adopted a phenomenological qualitative design. Twelve nurses in intensive care units assisting in Wuhan who experienced workplace violence during the COVID‐19 outbreak were selected using purposeful sampling. Data were collected through semi‐structured individual telephone interviews and analysed using Colaizzi's 7‐step method.

**Results:**

Analysis revealed three main categories including “Full of negative emotions”, “Facing challenges and danger” and “Coping methods”. The subjects experienced stress, fear, anger, helplessness, disappointment, sympathy and job burnout after suffering from workplace violence. The coping methods for workplace violence mainly included seeking support and help, escaping, making explanations, exercising tolerance and confronting the issue.

**Patient or Public Contribution:**

No patient or public contribution since nurses' experiences were explored.

## 1 INTRODUCTION

At present, coronavirus disease 2019 (COVID‐19) is still emerging and spreading in many countries around the world and has posed a remarkable challenge to world public health. According to data from Johns Hopkins University, in the 19 months since the outbreak, more than 5 million people worldwide have died of COVID‐19. With the ongoing global research and development of the COVID‐19 vaccine, people have increased confidence regarding the ability to overcome COVID‐19.

In the early stage of the COVID‐19 outbreak, the medical systems of various countries were greatly impacted by the lack of understanding of the virus, inadequate medical supplies, unclear therapeutic drugs, and the limited number of medical personnel. COVID‐19 not only poses a threat to people's lives and health but also causes a series of emotional reactions, such as pain and anxiety, due to the fear of the virus, isolation, work and school closures and inadequate resources for medical response (Pfefferbaum & North, 2020). In particular, the following groups are at increased risk for adverse mental health outcomes: people who contracted COVID‐19; those at heightened risk for the disease; and people with pre‐existing medical, psychiatric or substance abuse disorders (Pfefferbaum & North, 2020). COVID‐19 also exacerbates violence against health workers (Devi, 2020). Studies have shown (Najafi et al., 2018; Pompeii et al., 2015) that the altered mental status of patients is one of the main factors leading to workplace violence. Patients with a confirmed COVID‐19 diagnosis may experience fear of the consequences of infection with a novel, potentially fatal virus; symptoms of the infection; and adverse effects of treatment, which could lead to worsening anxiety and mental distress (Xiang et al., 2020). Additionally, the isolation of patients may result in anger, loneliness and other negative emotions, and the spread of misinformation about the virus (McKay et al., 2020), which may aggravate violence against medical staff. According to the International Committee of the Red Cross (ICRC), 611 incidents of violence, harassment or stigmatization against healthcare workers, patients and medical infrastructure during the COVID‐19 pandemic were recorded from February to July 2020; the actual number is likely to be much higher, and 67% of these incidents against healthcare workers (Devi, 2020).

Workplace violence (WPV) ISBN (2002) refers to the explicit or implicit challenge to the safety, well‐being and health of a worker caused by abuse, threat or attack in the normal working area, including psychological violence and physical violence, and can be divided into three forms: verbal assault, physical assault and sexual harassment. Among professionals, nurses have some of the highest incidences of WPV (Yang et al., 2018), which is a persistent and widespread problem worldwide. WPV negatively impacts nurses' mental health and happiness, and WPV is positively correlated with nurses' anxiety and depression levels (Zhao et al., 2018). In addition, WPV increases nurses' job burnout; reduces nurses' job satisfaction, confidence in their professional identity and sense of professional responsibility; increases turnover attempts; and greatly decreases the quality of nursing services (Chen et al., 2016; Najafi et al., 2018; Yang et al., 2018; Zhao et al., 2018). A survey of 1063 Chinese healthcare workers from 31 provinces and autonomous regions showed that 217 (20.4%) reported experiencing WPV during the COVID‐19 outbreak. Moreover, WPV was found to exert a detrimental effect on the mental health of Chinese healthcare workers, including their depression and anxiety, etc., during the COVID‐19 outbreak (Wang & Sang, 2020).

ICU nurses assisting in Wuhan not only work in an environment with a high risk of viral infection but also care for patients who need intensive care. In addition, the unfamiliar and specialized working environment and the high difficulty and intensity of the nursing workload make ICU nurses face much more pressure than nurses in mild or ordinary wards (Shen et al., 2020). Therefore, the exposure of ICU nurses to WPV may greatly negatively impact their mental health. At present, most studies have discussed the sources of stress and influencing factors of front‐line anti‐epidemic workers (Mo et al., 2020; Zheng et al., 2020), while few studies have focused on the impact of WPV on ICU nurses assisting in Wuhan. Therefore, through interviews with ICU nurses assisting in Wuhan who have suffered from WPV, this study aims to explore the psychological experience and coping methods of ICU nurses when dealing with WPV. Thus, the findings of this study may provide a reference for reducing the prevalence and negative impact of WPV towards health workers and formulating WPV emergency plans and work guidelines.

## 2 METHODS

### 2.1 Study design

Phenomenological research is suitable for understanding the essence of life experience which is understood from individuals give meaning to experiences towards a certain phenomenon and its applicability in understanding the voices of the participants (Salvador et al., 2021). Since the purpose of the study was to explore the psychological experience when dealing with WPV, a phenomenological qualitative study was conducted to provide in‐depth accounts of the psychosocial. This study selected nurses in Xiamen, China, who supported Wuhan during the initial period of the COVID‐19 outbreak as the research subjects.

### 2.2 Setting and sample

By utilizing a purposeful sampling method, we selected nurses caring for patients with COVID‐19 in the designated hospitals in Wuhan from February 2020 to March 2020. The inclusion criteria were as follows: (1) nurses who reported exposure to WPV during assistance in Wuhan; (2) nurses working in the ICU; (3) nurses who voluntarily participated in the study. The exclusion criteria were as follows: (1) providing support for less than 1 week; (2) not being directly involved in patient care.

### 2.3 Data collection

All participants were interviewed using a semi‐structured telephone interview, which is commonly used in phenomenological research to collect data (Castleberry & Nolen, 2018; Korstjens & Moser, 2018). Data collection was both private and anonymous. Before the interview commenced, the main content, purpose and significance of the interview were explained. After obtaining participants' informed consent, the interview was audio‐recorded taken on the main points. We developed the interview outline based on a review of relevant literature (Salvador et al., 2021), seeking three experts' opinions and selecting two nurses for preinterviews. The main interview questions posed to the participants were as follows: (1) Could you tell me about your experiences with violence while providing care for patients with COVID‐19? (2) How did you feel during and after this violent incident? (3) How do you deal with it, and could you describe or tell us about that experience?

Four authors (first, second, fourth and fifth) facilitated the interview sessions, two authors were responsible for the interview, another two authors were responsible for recording. In addition, the interviews were audio‐recorded with participants' permission and lasted from 30 to 60 min per session. When there was no new information derived from the interview, the data reached saturation. Data saturation was reached at the 12th interview. A trend was observed from the 9th to the 12th interview when emergent themes became repetitive. Seven participants worked in specialized hospitals, and five worked in general hospitals. The interviewee information is shown in Table 1. Each participant was interviewed once.

**TABLE 1:** Baseline characteristics of the interviewees (*n* = 12).

Characteristics	N (%) or mean ± SD	Median (range)
Gender		
Male	2 (16.7%)	
Female	10 (83.3%)	
Age (years)	29.83 ± 3.49	30 (24–36)
Education		
Undergraduate	8 (66.7%)	
Junior college	4 (33.3%)	
Occupation		
Nurse	2 (16.7%)	
Primary nurse	7 (58.3%)	
Supervisor nurse	3 (25.0%)	
Work experience (years)	7.17 ± 2.98	8 (2–11)
Department of the original unit		
Respiratory	3 (25.0%)	
Intensive medicine	3 (25.0%)	
Emergency	2 (16.7%)	
Oncology	2 (16.7%)	
Infectious diseases	1 (8.3%)	
Cardiology	1 (8.3%)	
Main categories		
Verbal violence	8 (66.7%)	
Physical violence	4 (33.3%)	

### 2.4 Ethical considerations

Prior to the study activities, human subjects approval was obtained through the REDACTED Scientific Ethics Committee (ethics code: REDACTED [2021]No.11). To avoid stress during working time, the interviews were scheduled in a convenient time for the nurses. Verbal consent with interviewees was obtained to reduce the risk of spreading disease through potentially contaminated paper and pens. Participants were guaranteed anonymity and confidentiality of information and audio files. They have the right to withdraw from the study at any time.

### 2.5 Data analysis

After ending the interview, the first author transcribed interviews verbatim in 24 h. The co‐authors checked the transcripts for accuracy. Colaizzi's seven‐step methodology (Wirihana et al., 2018) was used to guide data analysis. The seven steps include: (1) Read and re‐read all the participants' descriptions carefully. (2) Extract significant statements from each description. (3) Code meaningful points from these significant statements. (4) Organize these coded meaningful points into themes. (5) A detailed and unambiguous description was written. (6) Identify similar points of view and sublimate thematic concepts. (7) Return to participants for verification. Next, the data were coded independently and classified and themes were identified. When the individual researchers could not reach an agreement, the research team reached a consensus through discussion to ensure the accuracy of the results.

### 2.6 Trustworthiness and rigour

To guarantee the rigorousness of this qualitative study, four concepts were thoroughly considered: credibility, confirmability, dependability and transferability. To ensure credibility, the researchers who were involved in the data collection independently read the transcripts several times and coded them to analyse the data, this increased the rigour of this study by ensuring that multiple interpretations of the data were considered throughout the coding process. To improve confirmability, conflicting opinions on the content of a theme were discussed and resolved by a research group composed of four nurses with a master's in nursing and one nurse with a doctorate in nursing. The Consolidated Criteria for Reporting Qualitative Research Checklist and Standards for Reporting Qualitative Research were consulted to ensure the complete reporting of all relevant methods and findings. Regarding transferability, the characteristics of the research population and the research process were described clearly and accurately, to make it possible to follow the research path and key decisions made in the analysis.

## 3 RESULTS

### 3.1 Characteristics of the interviewees

In our study, we enrolled two male nurses and 10 female nurses aged between 24 and 36 years, with an average age of 29.83 ± 3.49. Their working experience ranged from 2 to 11 years, with an average of 7.17 ± 2.98 years. Table 1 outlines the baseline characteristics of the interviewees and types of violence (see Table 1).

### 3.2 Main themes

In addition, we explored the psychological experience and coping methods of the ICU nurses exposed to WPV. We identified three themes and nine subthemes that are summarized below (see Table 2).

**TABLE 2:** Example of stepwise analysis.

Theme	Subtheme	Quotations
Full of negative emotions	Anger	“Whenever the patient has something unpleasant, or if we are not doing well enough, they lose their temper. The patient is sometimes too unreasonable.”
		“In my busy work, the patient pushed me behind my back on purpose. I almost fell down. I was really angry and wanted to fight back.”
		“The patient spit indiscriminately. This is a crime. I really wanted to call the police and arrest him.”
	Helpless and disappointed	“The patient had not been cured, and we were also anxious for him. We hoped that he would be negative in the nucleic acid test as soon as possible. However, our ability was limited. It was useless for the patient to scold us.”
		“I felt very uncomfortable. I risked my life to support them. I wanted to do my best to help them, but she used these words to hurt me.”
	Understand and sympathize	“The patient was forced to be hospitalized and separated from his family because of COVID‐19, so it is understandable that the patient was in a bad mood, and the patient was only agitated for a while. I think psychological care is very important here.”
		“This patient has been living here for more than 2 months. His wife died because of the novel coronavirus. I see despair in his eyes.”
Facing challenges and danger	Fear of infection	Fear of getting infected with the disease
		Fear of transmitting the disease to colleagues
	Job burnout	“After being abused by the patient, I did not act as actively as before.”
		“Now I pay more attention to my own life safety and take personal protection more carefully. I think that I only need to follow the doctor's advice and provide the nursing care.”
		“I just want to go back to my workplace as soon as possible. The patients here do not seem to need me so much.”
	Difficult nursing	It's all serious patients, and I feel stressed
		Because I usually work in general ward, the nursing capacity of critically ill patients is still relatively insufficient
		Because of the lack of critical care ability, I am also worried that it will delay my colleagues
Coping methods	Seeking support and help	“After being scratched and threatened by patients, I rang the bell to receive support from other colleagues and leaders. They all supported me and made me feel safe.”
		“After being physically attacked by the patient, my colleagues quickly took me out of the violent environment, and security came to support me, so I was not afraid.”
		“After the violence happened, I told my closest colleague the cause and course of the incident and got the understanding and comfort of my colleagues. I felt much calmer.”
	Escape	“After being abused and threatened by the patient, I did not want to continue to care for the patient.”
		“I never want to recall this event again, never want to see this patient.”
	Explanation and tolerance	“If the patient's violent behaviour will not affect my life and health, I can tolerate it. After all, some of their family members were also infected with novel coronavirus and were quarantined or died. The patient is very poor.”
		“I patiently explained to the patients that we did not intentionally interrupt their rest and that we did so in the hope that they would recover soon.”
	Confrontation	“It was so infuriating. I could not control my emotions, and I quarrelled with the patient.”
		“I seriously warned the patient that if he continued this violent behaviour, I would call the police.”

#### 3.2.1 Full of negative emotions

##### Anger

Most of the interviewed nurses expressed anger. Nurses were burdened with excessively heavy workloads, wore uncomfortable protective clothing and risked potential COVID‐19 infection. Patients had a negative attitude towards nurses, did not cooperate with treatment and nursing, and even had physical conflicts with nurses. The nurses were very angry about the unreasonable demands and behaviours of patients.

“Whenever the patient has something unpleasant, or if we are not doing well enough, they lose their temper. The patient is sometimes too unreasonable.”

“In my busy work, the patient pushed me behind my back on purpose. I almost fell down. I was really angry and wanted to fight back.”

##### Helpless and disappointed

There was no clear treatment plan for COVID‐19. The nurses tried their best to provide the best care. Because patients' expectations were not met, they were angry with nurses. Some nurses felt helpless and disappointed.

“The patient had not been cured, and we were also anxious for him. We hoped that he would be negative in the nucleic acid test as soon as possible. However, our ability were limited. It was useless for the patient to scold us.”

“I felt very uncomfortable. I risked my life to support them. I wanted to do my best to help them, but she used these words to hurt me.”

##### Understand and sympathize

Regarding patients, patients had negative feelings due to the suffering associated with the disease and their separation from their family members. They sometimes could not control their tempers with nurses. The nurses could understand and sympathize with what the patients suffered. Therefore, the psychological care of patients should be strengthened.

“The patient was forced to be hospitalized and separated from his family because of COVID‐19, so it is understandable that the patient was in a bad mood, and the patient was only agitated for a while. I think psychological care is very important here.”

“This patient has been living here for more than 2 months. His wife died because of the novel coronavirus. I see despair in his eyes.”

#### 3.2.2 Facing challenges and danger

##### Fear of infection

In this study, most interviewees felt stress and fear after experiencing violence. The nurses worked in places with a high risk of viral infection. The violent behaviour of patients damaged the nurses' protective clothing and other protective measures, which could expose them and increase their risk of infection. Second, ICU nurses worked in closed wards. When a patient was violent, it was more difficult for the nurses to seek help and escape, and it was easy for patients to cause serious harm to the nurses.

“At that time, I was very scared. I quickly checked my protective suit to see if it was damaged. I was afraid of getting infected.”

“I was very scared at the time. I didn't know what the patient would do to me next, and I didn't know where to escape.”

##### Job burnout

The interviewees experienced a decline in work motivation within a week after violent events, which manifested as decreased communication with patients, reduced work efficiency, and increased expectation to return to their original units as soon as possible.

“After being abused by the patient, I did not act as actively as before.”

“Now I pay more attention to my own life safety and take personal protection more carefully. I think that I only need to follow the doctor's advice and provide the nursing care.”

“I just want to go back to my workplace as soon as possible. The patients here don't seem to need me so much.”

#### 3.2.3 Coping methods

##### Seeking support and help

Interviewees mentioned their gratitude for the support from colleagues, relatives, friends, and all sectors of society. The support of colleagues and leaders during violent incidents made nurses feel safe. After a violent incident, talking to family and friends could relieve stress.

“After being scratched and threatened by patients, I rang the bell to receive support from other colleagues and leaders. They all supported me and made me feel safe.”

"After being physically attacked by the patient, my colleagues quickly took me out of the violent environment, and security came to support me, so I was not afraid.”

“After the violence happened, I told my closest colleague the cause and course of the incident and got the understanding and comfort of my colleagues. I felt much calmer.”

##### Escape

Some nurses chose to escape. When violence occurred, they chose to leave the scene of violence. Afterwards, they said they did not want to recall the violent incident, which would cause them difficulty.

“After being abused and threatened by the patient, I did not want to continue to care for the patient.”

“I never want to recall this event again, never want to see this patient.”

##### Explanation and tolerance

The nurse could understand patients' impulsive behaviour and expressed sympathy for patients' suffering. Therefore, they chose to patiently explain the necessary care to patients and comfort them, hoping to receive their understanding and support.

“If the patient's violent behavior will not affect my life and health, I can tolerate it. After all, some of their family members were also infected with novel coronavirus and were quarantined or died. The patient is very poor.”

“I patiently explained to the patients that we did not intentionally interrupt their rest and that we did so in the hope that they would recover soon.”

##### Confrontation

A few nurses could not bear the violent behaviour of patients. When violence happened, they felt very angry and argued with patients. If a patient's violence was severe, they tried to solve the problem through legal means by calling the police.

“It was so infuriating. I couldn't control my emotions, and I quarreled with the patient.”

“I seriously warned the patient that if he continued this violent behavior, I would call the police.”

## 4 DISCUSSION

In the early stage of the outbreak of COVID‐19, China's public health system was seriously tested. In this context, medical workers shoulder arduous tasks as the main force in the fight against the epidemic (Sun et al., 2020). Studies have shown that during sudden natural disasters and infectious diseases, high‐intensity work will bring health threats and pressures to medical staff, who feel lonely and helpless (Khalid et al., 2016; Kim, 2018).

The incidence of workplace violence among healthcare workers is extremely high, and it has become one of the important factors threatening the personal safety of healthcare workers. Nurses are one of the professions most vulnerable to occupational violence, especially in the early stages of COVID‐19. The results of this study show that ICU nurses assisting in Wuhan had negative psychological experiences after they were exposed to WPV, which is consistent with the results of many studies (Najafi et al., 2018; Zhao et al., 2018; Zhang et al., 2018). Negative psychological feelings (such as stress, anxiety and depression) affect nurses' attention, understanding and decision‐making ability; obstruct epidemic prevention and control; and may have lasting adverse effects on nurses' overall health and seriously affect their work and quality of life. Therefore, measures should be actively taken to prevent and control the occurrence of violent incidents and bring awareness to the harm to health workers.

In addition to negative emotions, in the early stages of the COVID‐19 pandemic, the treatment plan was not clear, coupled with the special nature of work and the closed environment in the ICU, which increased nurses' work pressure, making their work full of challenges and dangers, and thus increasing job burnout. Friganović described that burnout syndrome is a serious problem for healthcare systems and affects almost all profiles of healthcare workers (Friganović et al., 2019). When facing professional violence, it is more important to focus on it. Therefore, active measures should be taken to prevent and control the occurrence of violent incidents. Nursing managers should pay attention to intervening from multiple aspects, such as strengthening training, violence prevention and psychological adjustment for high‐risk groups facing workplace violence, in order to prevent and reduce the occurrence of workplace violence, improve the quality and efficiency of nursing management.

In terms of personal coping, the results of this study indicate that nurses have adopted different coping styles in the face of occupational violence, including seeking support and assistance, escape and tolerance. Edward's system evaluation indicates that nurses who encounter WPV are more willing to seek empathy from other colleagues rather than seeking help from leaders, work units and professional institutions (Edward et al., 2014). Our research also confirms this point. Some nurses even dare not work or leave work alone after encountering WPV, avoid places and activities that trigger memories, and are shy of seeking help, attempting to temporarily gain psychological comfort through avoidance. Study recommends risk control in terms of environmental layout, security personnel and material allocation, administrative measures and work standards, and nurse training.

Many countries have taken actions and issued relevant laws and regulations (Devi, 2020; Indian government,  2020; The Supreme People's Court of the People's Republic of China, 2020), aiming at “zero tolerance” for violence against medical staff. In addition, it is necessary to increase investment in safety facilities in the intensive care unit and adopt mixed‐gender scheduling so that when a patient has a tendency for violence, the victim of violence can be helped in time. Professional psychologists need to assess the mental states of medical staff, strengthen psychological counselling and intervention, and improve the communication skills of medical staff to promote doctor/nurse–patient communication and improve patients' medical experience, which can effectively reduce the occurrence of violence. Finally, it is important to provide psychological support and intervention for medical workers who have experienced violence. Studies have shown that psychological intervention can eliminate or alleviate negative mental states among medical staff, strengthen their positive psychology and promote epidemic prevention and control work (Wang et al., 2020). Psychologists should provide personalized psychological support to medical staff who suffer from WPV, track their mental health status in a timely manner and provide psychological assessments and interventions to avoid serious posttraumatic stress disorder in medical staff in the future. Psychologists should strengthen the guidance regarding the psychological adjustment of medical staff, cultivate staff members' awareness of mental health management, and improve their self‐emotion adjustment ability. In addition, the social support system for medical staff should be strengthened, and family, friends, and colleagues should be encouraged to express concern and support for medical staff to alleviate the negative mood of the medical staff (Table 3).

**TABLE 3:** Strategies to prevent and control violence during COVID‐19 epidemic.

	Main measures
I. Ensure the safety of medical working environment and increase the sense of security of medical personnel	Promulgate relevant laws and regulations, with “zero tolerance” for violence Increase investment in safety facilities in wards: post notices on violent punishment for wounding health workers in accordance with the law and the telephone numbers of public security agencies in wards; instal cameras in wards; instal one‐key alarm bells for violence; strengthen safety patrols in wards, etc.; iii. Implement reasonable scheduling; include both male and female personnel in a work group
II. Strengthen the evaluation of patients' mental states and training in communication skills for health workers to promote doctor/nurse–patient communication	Equip wards with a professional psychologist Assess the patient's mental state in a timely manner Strengthen psychological counselling and intervention for patients iv. Strengthen guidance on communication skills for medical staff
III. Provide psychological support to medical staff to protect their physical and mental health	Provide medical staff with personalized psychological support, track their mental health status, and provide psychological evaluation and intervention Strengthen the guidance on the psychological adjustment of medical staff, cultivate awareness of mental health management, and improve self‐emotion regulation ability Strengthen social support systems for medical staff

## 5 LIMITATIONS

The main limitations of this study are as follows. First, only ICU nurses assisting in Wuhan from a designated support hospital in Hubei were interviewed; the small sample size and limited respondents may possibly have certain limitations. In the future, we should consider including nurses from different regions, different hospitals and different departments, to expand the sample size to extract more themes and provide response solutions to prevent and control violence during the COVID‐19 outbreak. Second, because the interview was conducted after the described violent incidents, the research participants may have had a certain recall bias when expressing their psychological experiences and coping styles when they were exposed to the violent incident. Third, this study did not thoroughly explore the predisposing factors of violence and its impact on the future work and lives of nurses. It is suggested that future research combine qualitative and quantitative research to further explore the factors that induce violence against health workers during epidemics and the far‐reaching impact of violence on medical staff. Finally, due to the uniqueness of the ICU working environment in Wuhan, the reference and transferability to other hospital ICUs or other departments may be limited.

## 6 CONCLUSION

During the COVID‐19 pandemic, health workers were exposed to life‐threatening risks that could seriously affect their physical and mental health and the progress of epidemic prevention and control. Therefore, there is an urgent need to address the violence against health workers. This article have a deep analysis of the psychology and coping styles of medical workers in Wuhan when facing occupational violence and provide reasonable suggestions. Medical staff should be strengthened to ensure their physical and mental health.

## 7 APPLYING RESEARCH TO OCCUPATIONAL HEALTH PRACTICE

This study demonstrates that the ICU nurses assisting in Wuhan had negative psychological experiences of workplace violence, which seriously affected their physical and mental health and nursing work. In order to reduce the occurrence of violence and strengthen the psychological assistance to nurses, this paper proposes the following suggestions. First, it is important to take intervention measures to ensure the safety of the medical working environment. Second, strengthen the evaluation of patients' mental states to identify possible violence as early as possible. In addition, strengthen psychological counselling and intervention for patients to promote doctor/nurse–patient communication. Finally, provide psychological support for health workers and strengthen their positive psychology.

## AUTHOR CONTRIBUTIONS

Guanhong Wu and Ying Lin drafted the manuscript and reviewed relative literatures, also participated in interviews and data collation. Xianghui Huang participated in the design of this study and revised the manuscript critically for important intellectual content. Jianshan Zheng and Mengmeng Chang participated in interviews and data collation.

## FUNDING INFORMATION

This study was supported by Guiding Project Xiamen Science and Technology Bureau (3502Z20189045).

## CONFLICT OF INTEREST STATEMENT

No conflict of interest has been declared by the authors.

## ETHICAL APPROVAL

Prior to the study activities, human subjects approval was obtained through the Xiamen Children's Hospital Scientific Ethics Committee (ethics code: Xiamen Paediatric Ethics Review [2021]No.11). Informed, written consent was obtained from participants prior to data collection. The authors promise that there will be no academic misconduct such as plagiarism, data fabrication, falsification and repeated publication.

## Data Availability

The data that support the findings of this study are available on request from the corresponding author. The data are not publicly available due to privacy or ethical restrictions.
